# Isoform switching and exon skipping induced by the DNA methylation inhibitor 5-Aza-2′-deoxycytidine

**DOI:** 10.1038/srep24545

**Published:** 2016-04-19

**Authors:** Xiao-Lei Ding, Xiaojing Yang, Gangning Liang, Kai Wang

**Affiliations:** 1 Co-Innovation Center for Sustainable Forestry in Southern China, College of Forestry, Nanjing Forestry University, Nanjing, Jiangsu, 210037, China; 2 Zilkha Neurogenetic Institute, University of Southern California, Los Angeles, CA 90033, USA; 3 Department of Urology, Keck School of Medicine, University of Southern California, Los Angeles, CA 90089, USA

## Abstract

DNA methylation in gene promoters leads to gene silencing and is the therapeutic target of methylation inhibitors such as 5-Aza-2′-deoxycytidine (5-Aza-CdR). By analyzing the time series RNA-seq data (days 5, 9, 13, 17) obtained from human bladder cells exposed to 5-Aza-CdR with 0.1 uM concentration, we showed that 5-Aza-CdR can affect isoform switching and differential exon usage (i.e., exon-skipping), in addition to its effects on gene expression. We identified more than 2,000 genes with significant expression changes after 5-Aza-CdR treatment. Interestingly, 29 exon-skipping events induced by treatment were identified and validated experimentally. Particularly, exon-skipping event in Enhancer of Zeste Homologue 2 (*EZH2*) along with expression changes showed significant down regulation on Day 5 and Day 9 but returned to normal level on Day 13 and Day 17. *EZH2* is a component of the multi-subunit polycomb repressive complex PRC2, and the down-regulation of exon-skipping event may lead to the regain of functional *EZH2* which was consistent with our previous finding that demethylation may cause regain of PRC2 in demethylated regions. In summary, our study identified pervasive transcriptome changes of bladder cancer cells after treatment with 5-Aza-CdR, and provided valuable insights into the therapeutic effects of 5-Aza-CdR in current clinical trials.

DNA methylation and histone modification play crucial roles in regulation of gene expression in mammalian developments as well as human diseases, such as cancer^1^,^2^. During tumorigenesis, the promoter regions of tumor suppressor genes could undergo abnormal hypermethylation, which lead to the silencing of these genes^3^,^4^,^5^. Moreover, transient exposure to low doses of DNA-demethylation agents can trigger durable antitumor effects in tumors^6^,^7^. Recently, clinical trials have been focused on investigating the possible utility of methylation inhibitors in solid tumors, either alone or in combination with other demethylation drugs^8^,^9^. Thus, reactivation of tumor suppressor genes by demethylation agents has become a possible and promising approach for cancer therapy.

Alternative splicing is closely associated with differentiation and development, and is a major source for protein diversity^10^. It enables cells to generate proteins of different coding sequences and functions from a single gene. Genome-wide approaches have revealed that tumorigenesis often involved large-scale alterations in alternative splicing^11^. Researchers also found that demethylation drugs could target transcribed regions, which suggest that the effects of demethylation drugs are not limited to the reactivation of promoters of silenced genes, but are prone to change exon recognition^6^,^12^,^13^. The demonstration that intragenic DNA methylation could affect elongation efficiency indicated that DNA methylation may facilitate exon inclusion^14^. A recent study further proved that intragenic DNA methylation modulated exon recognition, thus it is necessary to investigate the relationship between demethylation treatment and alternative splicing, which was generally overlooked in previous studies^15^.

DNA methyltransferases (DNMT) inhibitors, such as 5-azacytidine (5-Aza-CR) and 5-Aza-2′-deoxycytidine (5-Aza-CdR), were approved by the FDA for the treatment of myelodysplastic syndrome^16^,^17^. Therefore, a comprehensive understanding of how these demethylation drugs affect gene reactivation and alternative splicing is necessary for understanding their therapeutic effects and exploring new cancer therapies. In this study, we treated human bladder cell line UM-UC-3 with 5-AZA-CdR for 24 hours, then monitored expression changes at 5, 9, 13 and 17 days after treatment and employed deep RNA sequencing to analyze alterations in gene expression and alternative splicing. Additionally, we measured whole-genome methylation levels by the Illumina 450K methylation array at 5 and 17 days, to correlate with gene expression changes.

## Results

### Isoform expression changes induced by 5-Aza-CdR treatment

To explore the potential regulatory effects of 5-Aza-CdR, UM-UC-3 cells were treated with 0.1 uM 5-Aza-CdR for 24 hours, then collected at 5, 9, 13 and 17 days after treatment. Cells at the four time points together with untreated UM-UC-3 cells were then sequenced using paired-end Illumina RNA-Seq protocol, and two replicate experiments were performed for each sample. Approximately 20 Gb RNA-seq raw data for each replicate was generated after barcode removal and filtering of low-quality reads.

**Figure 1:**
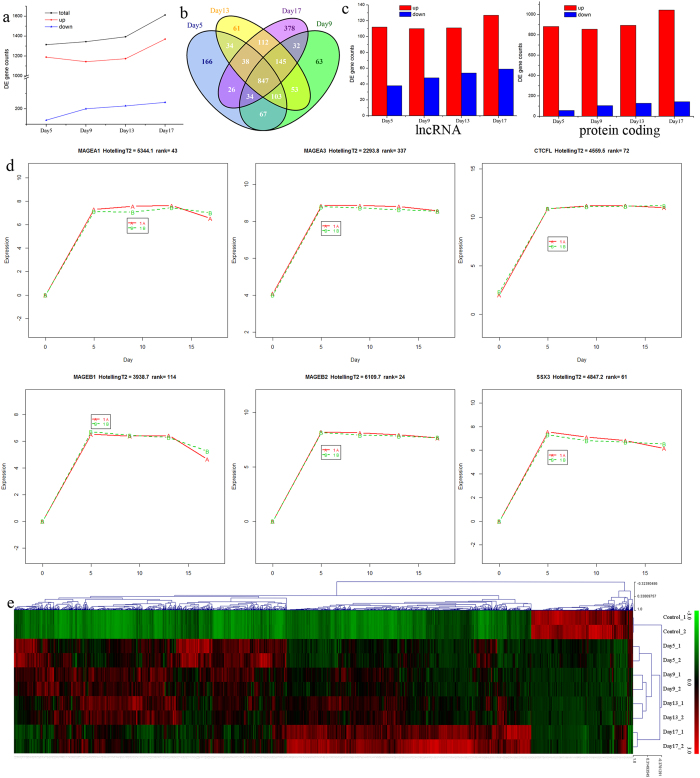
Dynamic transcriptome changes induced by 5-Aza-CdR treatment. (**a**) The number of differentially expressed genes across different time points. (**b**) Venn diagram showing overlapped differentially expressed genes found in each time point. (**c**) The number of up and down regulated genes found in the two RNA types: protein coding RNAs and lncRNAs. (**d**) Tumor suppressor gene expression pattern. (Y axis indicates normalized expression, X axis indicates different 5-Aza-CdR exposure time, A and B indicated two replicates) (**e**) Hierarchical clustering of differentially expressed genes.

RNA-seq data generated from untreated UM-UC-3 cells (control) and cells collected from four time points (Day 5, Day 9, Day 13 and Day 17) were aligned to human genome using Tophat2^18^ with GENCODE annotation (GRCh37.p13, GENCODE release 19). For all samples, we obtained more than 92% mapping ratio, which indicated high quality and reliability of the sequencing data. Differentially expressed (DE) genes were identified by comparing RNA-seq data obtained from each treatment with untreated cells. In total, 1315, 1344, 1393 and 1612 DE genes were found on Day 5, Day 9, Day 13 and Day 17, respectively (Fig. 1a). Among those DE genes, 847 of them were shared in all four time points (Fig. 1b). About 85% (Day 5: 90%, Day 9: 85%, Day 13: 84%, Day 17: 85%) DE genes were up regulated after 5-Aza-CdR treatment. Furthermore, the numbers of up and down regulated DE genes were positively correlated with the treatment time of 5-Aza-CdR and the most abundant DE genes were always found after 17 days treatment (Fig. 1a).

Based on RNA type annotation in GENCODE, about 72% and 12% of the DE genes are annotated as protein coding RNAs and lncRNAs, respectively (Fig. 1c, Supplementary Table S1). The numbers of protein coding RNAs and lncRNAs shared similar distributions as previously described DE genes across different time points (Fig. 1a,c). Here, we found that the expression of *HOTAIR* as well as several other tumor-suppressor lncRNAs such as *H19* and *MEG3* were reactivated after 5-Aza-CdR treatment for 5 days and maintained sustainable growth till Day 17^19^,^20^.

Previous studies on bladder cancer cells exposed to the 5-Aza-CdR for 8 days revealed that around 120 genes showed considerable changes^21^,^22^. Similarly, around 40 of previously reported DE genes were also identified in this study, while 30 of them were tumor suppressor genes (Fig. 1d, Supplementary Fig. S1), such as *MAGEA1, MAGEA3, MAGEA12, MAGEB1, MAGEB2, SSX1, SSX3* and *CTCFL*. We later examined the expression patterns of all DE genes and found that the expression for most genes increased with longer treatment time (Fig. 1e). Therefore, the effects of demethylation treatment on bladder cancer cells can be maintained for a long period of time.

### Detection of differentially expressed exons

**Figure 2:**
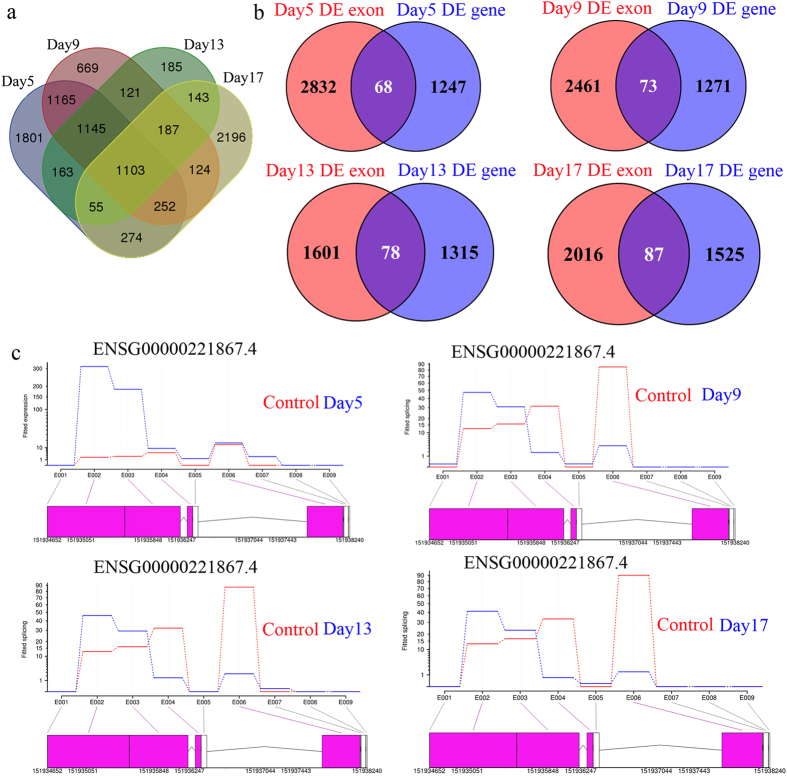
Summary of differentially expressed exons after 5-Aza-CdR treatment. (**a**) Venn diagram showing overlapped differentially expressed exons found in each time point. (**b**) Venn diagram showing overlapped genes identified as both differentially expressed genes and genes containing differentially expressed exons. (**c**) Visualization of differentially expressed exons found in *MAGEA3* (ENSG00000221867.4) after 5, 9, 13, and 17 days treatment (differentially expressed exons were highlighted in red).

Identification of DE exons can inform us on how the demethylation treatment affects exons recognition, and it can shed lights on the potential regulatory role of DNA methylation on alternative splicing. In total, 5958 (up: 3362, down: 2596), 4766 (up: 2364, down: 2402), 3102 (up: 1940, down: 1162) and 4334 (up: 2557, down: 1777) DE exons were identified on Day 5, Day 9, Day 13 and Day 17, respectively (P_adj_ < 0.05). Among them, 1103 exons were observed with significant changes across all time points (Fig. 2a). Unlike DE genes, the most abundant DE exons were identified on Day 5, followed with steady decrease on Day 9 and Day 13 and increased again on Day 17 (Fig. 2a). We also tried to measure the overlap between DE exons and DE genes. We found that 68, 73, 78 and 87 DE genes from Day 5, Day 9, Day 13 and Day 17 contained at least one DE exon (Fig. 2b). For instance, significant changes were found on exon 2, 3, 4 and 6 of tumor antigen *MAGEA3* across all time points, while *MAGEA3* itself was also known as a DE gene throughout 5-Aza-CdR treatment (Figs 2c and 1e). Interestingly, we also note that most DE genes do not contain any DE exon regardless of the exposure time (Fig. 2b). Thus, most of the genes may not be subject to isoform switching, despite that the overall expressions changed considerably. Functional analysis revealed that genes with DE exons were mainly associated with biological processes such as translational elongation, biopolymer methylation and DNA methylation. In summary, 5-Aza-CdR can not only induce changes in gene expression, but also exon-level changes for a small subset of DE genes.

### Identification of exon-skipping events

**Figure 3:**
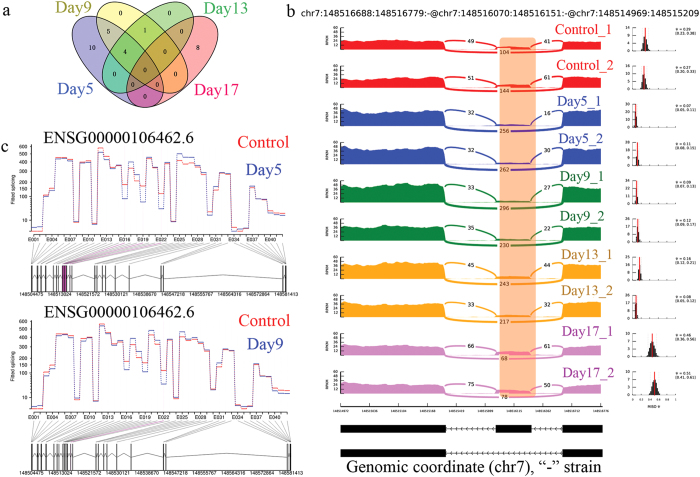
Exon recognition changes induced by 5-Aza-CdR treatment. (**a**) Venn diagram showing overlapped differentially expressed skipped exon events found in each time point. (**b**) Sashimi plot showing the expression changes of *EZH2* exon skipping (skipped exon was highlighted with orange rectangle) events at each time point. (ΔΨ > 0.15) (**c**) Visualization of differentially expressed exons found in *EZH2* after 5, 9, 13, and 17 days treatment (differentially expressed exons were highlighted in red).

We next tried to investigate whether demethylation treatment would induce alternative splicing in bladder cancer cells, since it has been reported in other cell lines^14^. Alternative splicing events can be classified into different types: skipped exon, mutually exclusive exon, alternative 5′ splice site, alternative 3′ splice site^23^. Here, we focused on exon-skipping events, since they are likely to be affected by methylation. DE exon-skipping were identified by comparing RNA-seq data from each time point with control using MISO^24^. Initially, we found 43, 26, 14 and 25 exon-skipping events, which showed considerable changes on Day 5, Day 9, Day 13 and Day 17 (Fig. 3a). To further validate our findings, we compared our DE exon-skipping results with previous DE exons. Intuitively, the DE exon-skipping events should involve some DE exons. We identified 28 (up:8 down:11), 10 (up:4 down:6), 5 (up:3 down:2) and 8 (up:5 down:3) overlapped DE exon-skipping events while 19 of them altered its coding sequence (Table 1). All five DE exon-skipping events found on Day 13 were included on Day 9 while four of them were also found on Day 5 except *RRBP1* (Table 1). In addition, nine exon-skipping events on Day 9 were also found on Day 5, again except *RRBP1*. However, eight exon-skipping events found on Day 17 together with ten events on Day 5 could not be observed at any other time points. Consequently, it seemed that exon-skipping events on Day 5 continued to show similar exon-skipping behavior on Day 9 and Day 13. After that, 17 days treatment resulted in eight other new DE exon-skipping events. One particular down regulated DE exon-skipping event was found in *EZH2* gene on Day 5 and Day 9 which was located in one of the *EZH2* transcripts annotated as being subject to nonsense-mediated decay by Ensemble (ENST00000483012.1) (Fig. 3b,c). *EZH2* belongs to PRC2/EED-EZH2 complex which catalyzes ‘Lys-27′ methylation of histone H3 and leads to transcriptional repression of the target genes. Therefore, this exon-skipping event is expected to cause the regain of functional *EZH2* gene.

**Figure 4:**
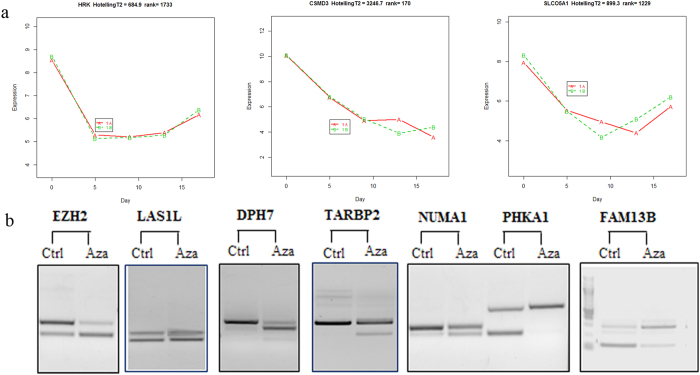
PcG (*EZH2*) mediated gene expression alteration and validation of exon-skipping events. (**a**) Differentially expressed PcG (*EZH2*) targeted genes induced by exon-skipping changes in *EZH2*. Aberrant exon recognition changes in *EZH2* found in Day 5 and Day 9 resulted in corresponding inhibition of targeted genes. (**b**) Experimental validations of seven differentially expressed exon-skipping events after 5-Aza-CdR treatment.

Finally, seven DE exon-skipping events were further validated by experimental verification. PCR results showed that bands with larger fragment sizes (fragment contained skipped exon) of *EZH2, LAS1L, DPH7, TARBP2* and *NUMA1* became lighter after 5-Aza-CdR treatment while bands with smaller fragment sizes (fragment without skipped exon) became darker, which indicated the down regulation of skipped exons. Among them, only *DPH7* was not identified with protein sequence change. Opposite observations can be found in another two up-regulated skipped exons of *PHKA1* and *FAM13B*, where expressions of fragment containing skipped exons were found to be increased after treatment (Fig. 4b, Table 1). Both of these two events resulted in protein sequence changes. In summary, 5-Aza-CdR treatments were also capable of inducing dynamic changes on exon-skipping events and most of the changes were consistent from Day 7 till Day 13. Some of these exon-skipping events may result in gene functional changes, such as truncated protein in *EZH2*.

### Comparison of DNA methylation and gene expression after 5-Aza-CdR treatment

To evaluate whether DNA methylation is responsible for our findings above, we assayed the UM-UC-3 cells treated with 5-Aza-CdR for 5 and 17 days, by the Illumina 450K methylation array. In total, 18,906 and 18,740 genes were demethylated after 5-Aza-CdR treatment on Day 5 and Day 7, respectively. Meanwhile, previous results indicated that 1,315 and 1,612 DE genes were identified at the same time points. Among them, over 60% DE genes (mostly protein-coding genes) showed demethylation after drug treatment, while the rest were mostly non-coding RNAs such as lncRNAs and pseudogenes (Supplementary Fig. S2). Furthermore, among the 28 genes with exon-skipping events, 22 were demethylated after drug treatment and 19 of them showed demethylation within their gene bodies (Table 1). The observation is consistent with the hypothesis that methylation in the gene body may have an impact on splicing^25^. Altogether, these results provided concrete evidence that 5-Aza-CdR treatment can cause isoform switching and exon skipping events.

## Discussion

Low dosage (0.1 uM) of 5-Aza-CdR treatment was widely used in current clinical trials because of its high demethylation efficacy and low toxicity. In addition, it has been shown that demethylation is not the main driving force in phenotypic changes by high dosage of 5-Aza-CdR^26^,^27^, which is why our study on 5-Aza-CdR only focused on low dose treatment. The efficiency of 5-Aza-CdR in regulating DNA methylation changes indicated that drug-induced demethylation may be an effective therapeutic intervention in cancer, since some tumor suppressor genes were reactivated as reported above (Fig. 1e). Furthermore, the number of DE genes continued to increase on Day 13 and Day 17 and there were 847 DE genes which are shared through all time points (Fig. 1a,b). Among all DE genes, we observed expression changes of a small set of lncRNAs. LncRNAs are known to be involved in cancer progression, and received more and more attention recently because of their potential as biomarkers and novel therapeutic targets for cancer^28^. For instance, an oncogenic lncRNA *HOTAIR* interacts with PRC2 complex to repress the *HOXD* locus and a new study indicates that *HOTAIR* is over-expressed in breast tumors^28^,^29^. All the results suggested that the gene reactivation induced by 5-Aza-CdR appeared to be progressive and long-lasting, maintaining for at least 17 days, which also supported by recent studies^6^,^7^,^30^.

Previous studies showed that exons are more highly methylated than introns and the degree of methylation differs at exon–intron boundaries^31^. Most recent research further showed that DNA methylation had an effect in regulating exon skipping^15^.Consequently, DNA methylation can possibly mediate RNA splicing^32^,^33^. In this research, we also found a small set of exon-skipping events induced by 5-Aza-CdR (Table 1). Combined with our previous results, it seemed that the longest exposure time to 5-Aza-CdR always produced more DE genes, DE exons and exon-skipping events, which may lead to alteration of gene function. However, due to tissue-specific DNA methylation pattern, we expect that DE genes (and isoform switching events) may be varied between different cell types^25^.

Unlike other inhibitors targeted to inhibit the overall expression of a polycomb group (PcG) protein *EZH2*^34^,^35^,^36^,^37^, we found one skipped-exon event (chr7:148516070-148516151) from *EZH2*, which showed significant down-regulation after 5 and 9 days post 5-Aza-CdR treatment (Fig. 3b,c). This down-regulated exon was known as a poison exon which could lead to the nonsense-mediated decay of *EZH2* by introducing premature termination codon^38^. Thus, the exclusion of this poison exon could cause the regain of functional *EZH2*. *EZH2* concerts with other proteins (*EED, SUZ12* and *RBBP4*) to form the PRC2, which can initiate polycomb-mediated gene repression^39^. PcG marks genes that are prone to cancer-specific DNA hypermethylation and the remaining of PcGs may lead to the exclusion of DNA methylation for certain gene^40^,^41^,^42^. On the other hand, demethylation may cause regain of PRC2 in demethylated regions^43^. Interestingly, we found that a small set of PRC2 targeted genes (*HRK*, *CSMD3* and *SLCOSA1*) were inhibited on Day 5 and Day 9 (Fig. 4a)^44^. These particular genes were validated to be unable to gain chromatin accessibility despite the promoter demethylation in our previous findings^43^. Perhaps the down-regulation of *EZH2* exon-skipping event on Day 5 and Day 9 could lead to the regain of PRC2 while the recovery of exon-skipping in Day 13 and Day 17 could cause the loss of PRC2^45^. In general, permanent gene silencing require DNA methylation coupled with PRC2, such as *MYT1* and *CNR1*^46^. However, our results demonstrated that DNA methylation may play a key role to silence *CNR1* in UM-UC-3 cell line, since after demethylation treatment the expression of *CNR1* was activated regardless of the exon change in *EZH2*. In addition, another 9 genes marked with PcG and reported to be hypermethylated in cancer showed significant changes throughout the demethylation treatment (Supplementary Fig. S3)^40^. Thus, reactivation of expression for those genes may be caused by demethylation treatment by 5-Aza-CdR. Other exon-skipping events were found to be associated with tumor necrosis factor-mediated signaling pathway (*KRT8*) and neuroblast proliferation (*ASPM* and *NUMB*)^47^,^48^. Based on our results and previous studies, the methylation that occurred in transcribed regions may contribute to nucleosome destabilization and reduced efficiency of splicing, while inhibition of DNA methylation led to aberrant splicing^6^,^15^, since all these genes with DE exon-skipping events did not show significant changes on transcript level (Fig. 4b).

Take together, our study demonstrated that DNMT inhibitor 5-Aza-CdR can alter expression patterns of many genes on both the isoform and exon level. More importantly, we showed that DNA methylation was associated with alternative splicing and 5-Aza-CdR was able to change the exon-skipping in *EZH2*. This study provides valuable information on how demethylation drugs affect bladder cancer cells, thus shedding light on ongoing and future clinical trials that evaluate demethylation drugs.

## Materials and Methods

### Tissue Culture and 5-Aza-CdR Treatment

UM-UC-3 cells were procured from American Type Culture Collection (ATCC), and were used for all *in vitro* studies. No human subjects were used in the study. The methods were carried out in accordance with approved guidelines, and the experimental protocols were approved by USC.

UM-UC-3 cells were maintained in MEM medium, supplemented with 10% fetal bovine serum and 1% penicillin/streptomycin. UM-UC-3 cells were treated with 0.1 uM of 5-Aza-CdR (Sigma-Aldrich). The medium was changed 24 hr later. RNA was harvested 5, 9, 13 and 17 days after drug treatment and was extracted using Direct-zol™ RNA MiniPrep (Zymo).

### Library preparation and Illumina sequencing

The RNA-seq library was generated using Illumina TruSeq RNA Sample Preparation kit and was sequenced using 100 bp paired-end model at the University of Southern California Epigenome Center according to the manufacturer’s specifications. Generally, 20 Gb raw RNA-seq data for each time point was obtained and two replicates for each time point were sequenced.

### Bioinformatics analyses

Raw RNA-seq data were first assessed by Fastqc (http://www.bioinformatics.babraham.ac.uk/projects/fastqc/). Adapters were then removed by cutadapt package (https://code.google.com/p/cutadapt/). After that, filtered reads were aligned to the human genome by TopHat2 using Gencode annotation (GRCh37.p13, GENCODE release 19)^18^. Normalized read count for all genes were obtained using HTseq and subsequent DE genes were identified by DESeq^49^,^50^ (p_adj_ < 0.01,fold change > 10). Individual gene expression was analyzed using “timecourse” package from R (http://www.bioconductor.org/packages/release/bioc/html/timecourse.html). Hierarchical clustering was performed by MEV package (http://www.tm4.org/mev.html). All DE genes were then imported into the DAVID website for functional and pathway enrichment analysis^51^. DE exons were identified by R package DEXSeq which focused on finding differential exon usage using RNA-seq exon counts between samples with different experimental designs (p-value < 0.05)^52^. DE exons can be visualized using built in DEXSeq plot function. MISO served as the main tool for exploring exon-skipping events^53^. Pre-build GFF3 annotation files were downloaded from MISO website (http://miso.readthedocs.org/en/fastmiso/annotation.html) and customized Perl scripts were used to screen and located the genes containing exon-skipping events. Differentially expressed exon-skipping events were detected with “compare_miso–compare-samples” option and further filtered with “–num-sum-inc-exc 10–delta-psi 0.15–bayes-factor 10” parameters according to MISO documentation (http://miso.readthedocs.org/en/). All exon-skipping events were visualized by sashimi_plot (http://miso.readthedocs.org/en/fastmiso/sashimi.html).

### Illumina Infinium HM450 DNA methylation assay

The Infinium DNA methylation assay was performed at the University of Southern California Epigenome Center according to the manufacturer’s specifications. The HM450 BeadChip examines the DNA methylation status of 482,421 CpG sites, covering 99% of RefSeq genes and intergenic regions. The DNA methylation level is reported as a beta value, ranging from 0 (not methylated) to 1 (fully methylated). The delta beta value 0.2 was selected as cut-off to identify genes with demethylation after drug treatment.

### Experimental validation of identified skipped-exon events

RT-PCR primers were designed to amplify upstream and downstream exons if the potential skipped exons were less than 200 bp. If the potential skipped exon is larger than 200 bp, which would introduce bias selection for PCR amplification, a forward primer was designed at the potentially skipped exon with similar amplification efficiency. Primer sequences are all available upon request.

## Additional Information

**Accession codes:** All RNA-Seq sequences were deposited in the NCBI SRA database with accession number: SRP063667.

**How to cite this article**: Ding, X.-L. *et al.* Isoform switching and exon skipping induced by the DNA methylation inhibitor 5-aza-2'-deoxycytidine. *Sci. Rep.* **6**, 24545; doi: 10.1038/srep24545 (2016).

## Supplementary Material

Supplementary Information

## Figures and Tables

**Table 1:** List of exon-skipping events identified by MISO and DEXSeq.

Gene name	Changes induced by 5-Aza-CdR				Genomic coordinate (skipped exon)	Alter protein sequence	Gene body demethylation
	Day5	Day9	Day13	Day17			
YDJC	down	N/A	N/A	N/A	chr22:21983299-21983476	no	yes
DLGAP5	down	down	N/A	N/A	chr14:55615312-55615402	yes	yes
LAS1L	down	down	N/A	N/A	chrX:64744444-64744494	yes	no
EZH2	down	down	N/A	N/A	chr7:148516070-148516151	yes	yes
TARBP2	down	N/A	N/A	N/A	chr12:53898919-53899046	yes	yes
UAP1	down	down	N/A	N/A	chr1:162562525-162562572	yes	no
NUMA1	down	N/A	N/A	N/A	chr11:71723447-71723488	yes	yes
CSNK1G3	down	N/A	N/A	N/A	chr5:122941033-122941056	yes	yes
KRT8	down	down	down	N/A	chr12:53343240-53343362	no	yes
DPH7	down	N/A	N/A	N/A	chr9:140470761-140470854	no	no
MCM3	down	down	down	N/A	chr6:52130033-52130183	yes	yes
INSIG1	up	N/A	N/A	N/A	chr7:155095535-155095605	yes	yes
ACYP1	up	N/A	N/A	N/A	chr14:75528387-75528465	yes	yes
ORMDL1	up	up	N/A	N/A	chr2:190647740-190647849	no	no
FAM13B	up	N/A	N/A	N/A	chr5:137354644-137354835	yes	no
PHKA1	up	up	up	N/A	chrX:71840575-71840751	yes	no
IKBIP	up	up	up	N/A	chr12:99028074-99028191	yes	yes
ASPM	up	N/A	N/A	N/A	chr1:197069561-197074315	yes	yes
NUMB	up	N/A	N/A	N/A	chr14:73745989-73746132	yes	yes
RRBP1	N/A	up	up	N/A	chr20:17660644-17660720	no	yes
DIS3	N/A	N/A	N/A	up	chr13:73355427-73355494	no	no
HRAS	N/A	N/A	N/A	up	chr11:533277-533358	yes	yes
SRP9	N/A	N/A	N/A	up	chr1:225974564-225974687	yes	yes
PPHLN1	N/A	N/A	N/A	up	chr12:42768440-42768535	no	yes
TIAL1	N/A	N/A	N/A	up	chr10:121336359-121336417	no	yes
SMIM7	N/A	N/A	N/A	down	chr19:16764846-16764936	no	no
SCARB1	N/A	N/A	N/A	down	chr12:125267229-125267357	yes	yes
CTC-429P9.4	N/A	N/A	N/A	down	chr19:16764846-16764936	no	no
