# Supplementary material for: Isoform switching and exon skipping induced by the DNA methylation inhibitor 5-Aza-2′-deoxycytidine
Source: Sci Rep. 2016 Apr 19;6:24545. doi: 10.1038/srep24545 (PMC4835787; doi:10.1038/srep24545)
Supplement: Supplementary Information [file srep24545-s1.pdf]

Isoform switching and exon skipping induced by the DNA methylation inhibitor 5-aza-2'-deoxycytidine

Xiao-Lei Ding[1,2], Xiao-Jing Yang[3], Gangning Liang[3]*, Kai Wang[2]*

[1]Co-Innovation Center for Sustainable Forestry in Southern China, College of Nanjing Forestry University, Nanjing, Jiangsu, China

[2]Zilkha Neurogenetic Institute, University of Southern California, Los Angeles, CA 90033

[3]Department of Urology, Keck School of Medicine, University of Southern California, Los Angeles, CA 90089, USA

*Correspondence: gliang@usc.edu (GL), kaiwang@usc.edu (KW)

Supplementary Tables:

Supplementary Table S1. Detailed annotation results of all differentially expressed genes after 5-Aza-CdR treatment.

Day5	Total	Up	Down
lncRNA	150	112	38
sense intronic	6	5	1
pseudogene	101	92	9
sense overlapping	3	3	0
processed transcript	16	15	1
antisense	92	75	17
miRNA	2	1	1
protein coding	940	882	58
misc_RNA	5	4	1

Day9	Total	Up	Down
polymorphic pseudogene	1	1	0
lncRNA	158	110	48
sense intronic	7	4	3
pseudogene	105	87	18
sense overlapping	6	4	2
processed transcript	16	15	1
antisense	82	62	20
miRNA	3	1	2
protein coding	961	856	105
misc_RNA	5	4	1

Day13	Total	Up	Down
polymorphic pseudogene	1	1	0
lncRNA	165	111	54
sense intronic	3	3	0
pseudogene	102	84	18
sense overlapping	8	5	3
processed transcript	14	12	2
antisense	72	59	13
TR_V_gene0	1	1	0
miRNA	1	1	0
protein coding	1022	894	128
misc_RNA	4	3	1

Day17	Total	Up	Down
polymorphic pseudogene	2	2	0
lncRNA	186	127	59
sense intronic	4	2	2
pseudogene	113	95	18
snRNA	1	1	0
sense overlapping	6	3	3
processed transcript	16	14	2
antisense	94	79	15
protein coding	1187	1044	143
miRNA	1	1	0
misc_RNA	2	2	0

Supplementary Figures

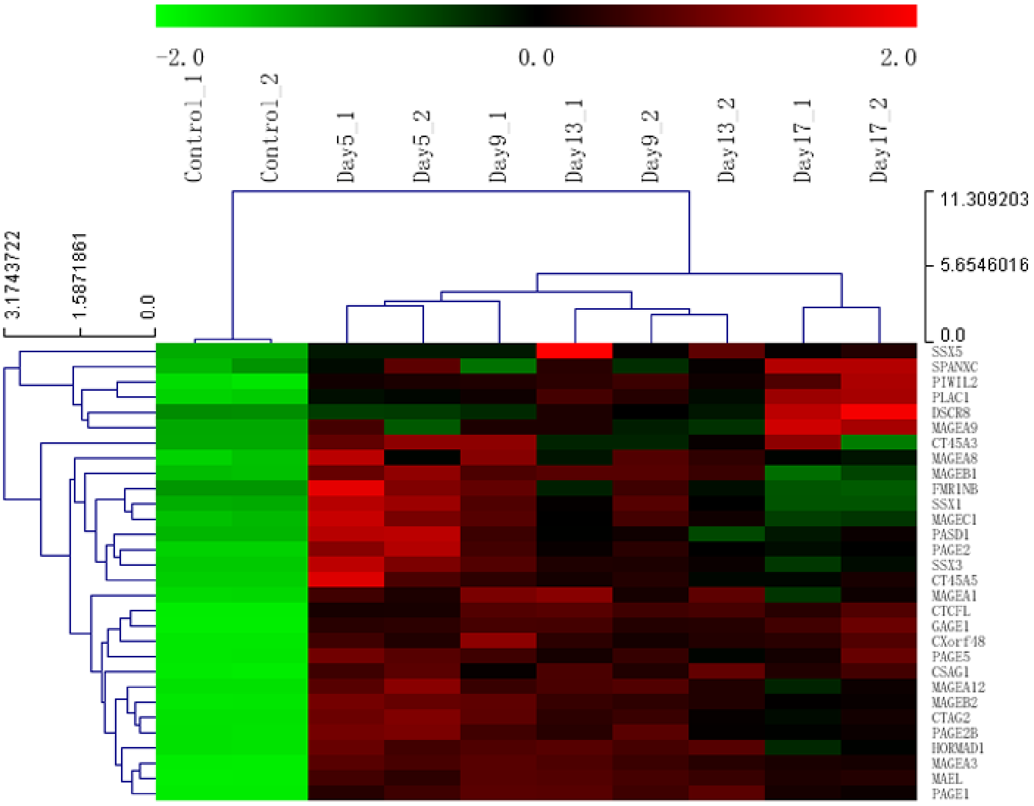

Supplementary Figure S1. Hierarchical clustering of differentially expressed tumor associated antigens after 5-Aza-CdR treatment.

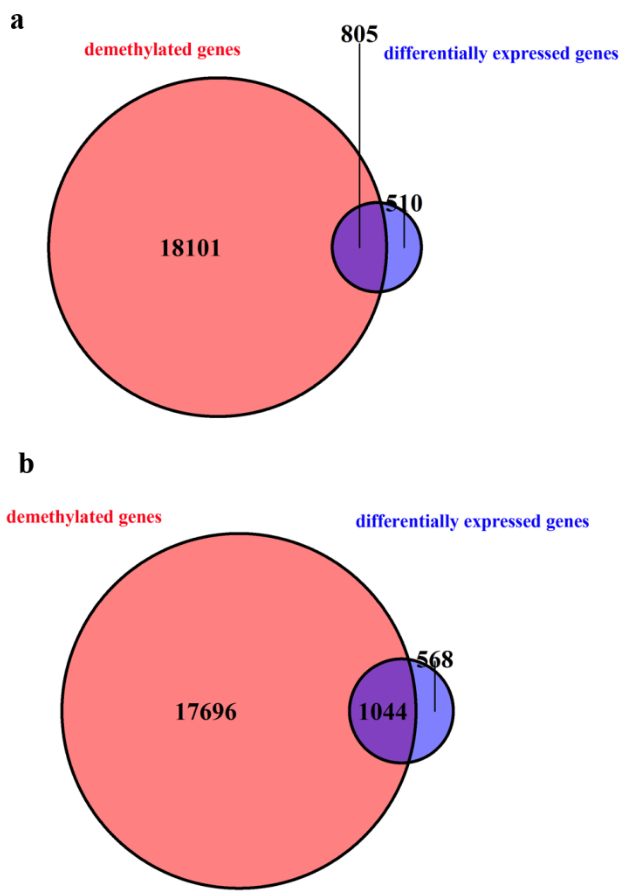

Supplementary Figure S2. Comparison of whole-genome DNA methylation levels with gene expression changes after 5-Aza-CdR treatment. (a) 5-Aza-CdR treatment for 5 days. (b) 5-Aza-CdR treatment for 17 days.

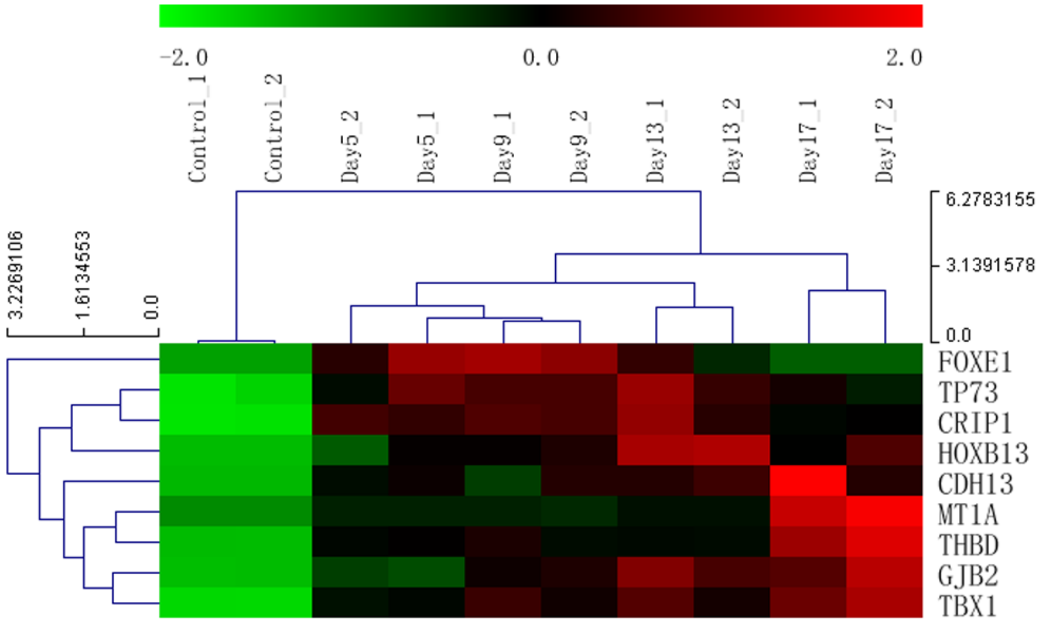

Supplementary Figure S3. Hierarchical clustering of 9 differentially expressed genes which marked with PcG (*EZH2*) and reported to be hypermethylated in cancer.
